# Evaluation of clinical and laboratory findings in MIS-C patients associated with COVID-19: An experience from the Northwest of Iran

**DOI:** 10.1371/journal.pone.0313843

**Published:** 2024-11-21

**Authors:** Mina Farshidgohar, Sonia Oveisi, Samira Dodangeh, Fatemeh Fawzi, Faezeh Maleki Sanjani, Alireza Razzaghi, Hossein Teimouri, Gerson Nakazato

**Affiliations:** 1 Clinical Research Development Unit of Advanced Medicine, Qazvin University of Medical Sciences, Qazvin, Iran; 2 Student Research Committee, Qazvin University of Medical Sciences, Qazvin, Iran; 3 Research Institute for Prevention of Non-Communicable Diseases, Children Growth Research Center, Qazvin University of Medical Sciences, Qazvin, Iran; 4 Research Institute for Prevention of Non-Communicable Diseases, Social Determinants of Health Research Center, Qazvin University of Medical Sciences, Qazvin, Iran; 5 Medical Microbiology Research Center, Qazvin University of Medical Sciences, Qazvin, Iran; 6 Department of Microbiology, Laboratory of Basic and Applied Bacteriology, Center of Biological Sciences, State University of Londrina, Londrina, Brazil; Children’s National Hospital, George Washington University, UNITED STATES OF AMERICA

## Abstract

This study aimed to evaluate the range of clinical and laboratory findings of multisystem inflammatory syndrome in children (MIS-C) with COVID-19 in a tertiary children’s hospital in Northwest Iran during 2020–2022. According to the CDC guidelines, this cross-sectional study included 300 pediatric patients diagnosed with MIS-C. Data were collected retrospectively from medical records, focusing on symptoms, organ involvement, laboratory findings, and outcomes. Statistical analysis was performed using SPSS software, with significance set at p-values <0.05. The study population had a median age of 3 years, with a slight male predominance (57.3%). The most affected systems in MIS-C disease were hematological (87%), gastrointestinal (85%), and respiratory (67%). Laboratory analysis highlighted elevated inflammatory markers such as D-dimer (83.3%), ferritin (71.4%), and CRP (49.7%). Abnormal urinalysis was observed in 151 patients (50.3%), with glucosuria in 83 cases (27.7%) and proteinuria in 29 cases (9.7%). The study found a significant correlation between cardiovascular issues and elevated blood platelets, ESR, CRP, and troponin levels (P ≤ 0.01) but not with ferritin, albumin, or D-dimer levels. Also, the examination of disease outcomes in this study revealed that 81.7% of MIS-C patients were isolated during their hospital stay, 18.3% needed ICU care, and 1% died in hospital. We have presented an experience with distinct clinical and laboratory manifestations in MIS-C. Given the lower median age in this study compared to previous studies, reporting clinical and laboratory manifestations of MIS-C in pediatrics with a younger age is valuable for the diagnosis and treatment course. Some laboratory factors were risk factors for cardiovascular involvement, and consequently, echocardiography is recommended in MIS-C patients with these laboratory indications. Given the lack of a specific diagnostic test for this emerging disease, studies focusing on investigating clinical symptoms and findings are valuable.

## Introduction

Since December 2019, the global outbreak of Coronavirus disease-2019 (COVID-19), which is caused by the severe acute respiratory syndrome coronavirus 2 (SARS-CoV-2), has unfolded worldwide [1–3]. Although data gathered during the initial stages of the epidemic showed that fever, dry cough, fatigue, and myalgia were the predominant symptoms of COVID-19, more serious complications such as acute respiratory distress syndrome (ARDS) and even significant mortality were seen in patients [4]. Underlying conditions, such as cardiovascular diseases, have been identified as significant risk factors for increased severity and poorer prognosis in COVID-19 patients [5]. Furthermore, clinical markers, including elevated cytokine levels of IL-6 and IL-8, function as biomarkers signifying a poor prognosis. Conditions such as primary immunodeficiency and cytokine storms significantly heighten the risk and severity of COVID-19 complications [6].

Recent studies have highlighted that children, similar to adults, can experience significant long-term effects from COVID-19. Pediatric Long COVID-19 can lead to substantial disabilities, even in previously healthy children, and encompasses a range of symptoms from mild to severe infections [7]. During the apex of the COVID-19 pandemic, hundreds of children and adolescents developed Kawasaki disease (KD)-like symptoms such as fever, conjunctivitis, peripheral edema, and gastrointestinal issues like diarrhea, vomiting, and abdominal pain in Europe and the United States in the spring of 2020, and a few of them died from this disease [8]. In these patients, inflammatory indicators (erythrocyte sedimentation rate [ESR], C-reactive protein [CRP], procalcitonin, ferritin, interleukins [ILs], etc.) and neutrophils were also significantly increased [9]. Similar cases subsequently appeared worldwide, and this potentially life-threatening condition was named multisystem inflammatory syndrome in children (MIS-C) by the Centers for Disease Control and Prevention (CDC) and the World Health Organization (WHO) [10]. The disease usually begins 2–4 weeks after SARS-CoV-2 infection in children 6 months to 17 years of age [11]. This syndrome is of particular concern because most cases have been reported in children with no underlying health conditions [12]. This unexpected presentation in previously healthy children challenges current understanding and highlights the unpredictable nature of SARS-CoV-2, which can lead to severe complications even in those without prior health issues [13]. Therefore, it is crucial to develop effective diagnostic and treatment strategies to manage this condition in a population previously thought to be at lower risk.

Diagnosis of MIS-C cases is based on six main elements, including clinical features and laboratory findings [14], as follows: 1) age of children, 2) persistence of fever, 3) elevated inflammatory biomarkers, 4) symptoms and/or signs of organ dysfunction, 5) lack of an acceptable alternative diagnosis, and 6) evidence of COVID-19 or exposure to COVID-19. MIS-C may have shared features with KD, including mucocutaneous involvement, conjunctivitis, lymphadenopathy, and elevated inflammatory markers. Since MIS-C was identified, discussions about differentiating MIS-C from KD have continued among professionals worldwide [15,16].

Therefore, the geographic, genomic, and ethnic distribution of children with MIS-C should be considered to identify accurate diagnostic criteria and optimal treatment guidelines. More comprehensive studies are needed to enhance insight into MIS-C symptoms, treatments, outcomes, and preventive measures in children.

Considering the new nature of this disease and the lack of epidemiological investigation in Iran, we conducted this study to evaluate the range of clinical and laboratory symptoms of MIS-C in children with COVID-19 in a tertiary Children’s Hospital during 2020–2022.

## Materials and methods

A cross-sectional study was carried out in an isolation unit and the Pediatric Intensive Care Unit (PICU) at Children’s Hospital of Qazvin, Iran, from March 20, 2020, to March 20, 2022, using records of patients with MIS-C. Data were accessed for research purposes during the study period from October 15, 2022, to March 11, 2023.

The current investigation was carried out in accordance with the principles and recommendations outlined in the Helsinki Convention. Utmost confidentiality was maintained for all gathered information. The ethics committee of Qazvin University of Medical Sciences approved this project, assigning it the ethics code IR.QUMS.REC.1400.488.

Considering that the minimum prevalence of MIS-C in hospitalized children is 60% [17], the prevalence of 60% was considered in this study. The required sample size for this study was determined to be 266 patients based on the following formula.


$$N=\frac{z_{1-\frac{\alpha }{2}}^{2}*P(1-P)}{d^{2}}=266$$

α=0.05,P=0.6,q=0.4,d=0.06

The study’s inclusion criteria encompassed patients with MIS-C who were hospitalized from 2020 to 2022. Furthermore, the patients were required to undergo a Reverse transcription polymerase chain reaction (RT-PCR) test, an antibody test, or an antigen test for SARS-CoV-2 within the first 48 hours of their admission to intensive care to confirm a current or recent infection. Exclusion criteria consisted of patients unable to receive a MIS-C diagnosis due to incomplete medical history, patients infected with COVID-19 but lacking fever with involvement of at least two body organs (thus not meeting the MIS-C definition), and patients meeting the MIS-C definition but having an underlying disease or concurrent infections (e.g., positive urine, stool, or sputum cultures for bacterial or fungal agents), leading to their exclusion from the study.

The diagnosis of MIS-C was determined based on the CDC health guidelines, which involved reviewing the medical records of individuals under the age of 21. These records were assessed for the presence of fever and the involvement of at least two organs, such as the heart, lungs, brain, skin, eyes, digestive system, or blood. Additionally, the patients’ COVID-19 PCR test results, positive serological test, or history of contact with a positive individual within the past four weeks were also considered [8]. The instrument utilized in this investigation was a checklist comprising clinical and paraclinical manifestations that were assessed in individuals diagnosed with multisystem inflammatory syndrome.

### Statistical analysis

Data were analyzed using SPSS version 26 software. The Kolmogorov-Smirnov test was used to check the normality or non-normality of quantitative data distribution. The frequency and percentages for qualitative variables and the mean and standard deviation for quantitative variables with normal distribution were calculated. The chi-square test was used to check the relationship between qualitative variables. P-values <0.05 were considered significant. The raw data supporting this analysis are available in the S1 File.

## Results

In the current study, the files of 300 patients fitting the definition of MIS-C were evaluated. The age range was from one month to 12 years, with a median age of 3 years. Of the patients, 42.7% were female (128 patients) and 57.3% were male (172 patients).

In this study, we also categorized the age stages into three groups: neonatal and infancy (from birth to 1 year), childhood (from 1 year to 10 years), and adolescence (from 10 years to 18 years). Of the patients, 168 (56%) belonged to the childhood age group, 96 (32%) were in the neonatal and infancy age group, and 36 (12%) were in the adolescence age group.

Also, 185 patients (61.7%) reported contact with a COVID-19-positive individual within four weeks before hospitalization. SARS-CoV-2 RT-PCR was positive in 17 patients (5.7%), while the rest (98 individuals, 32.6%) had both a positive contact history and positive SARS-CoV-2 RT-PCR results.

**Fig 1:**
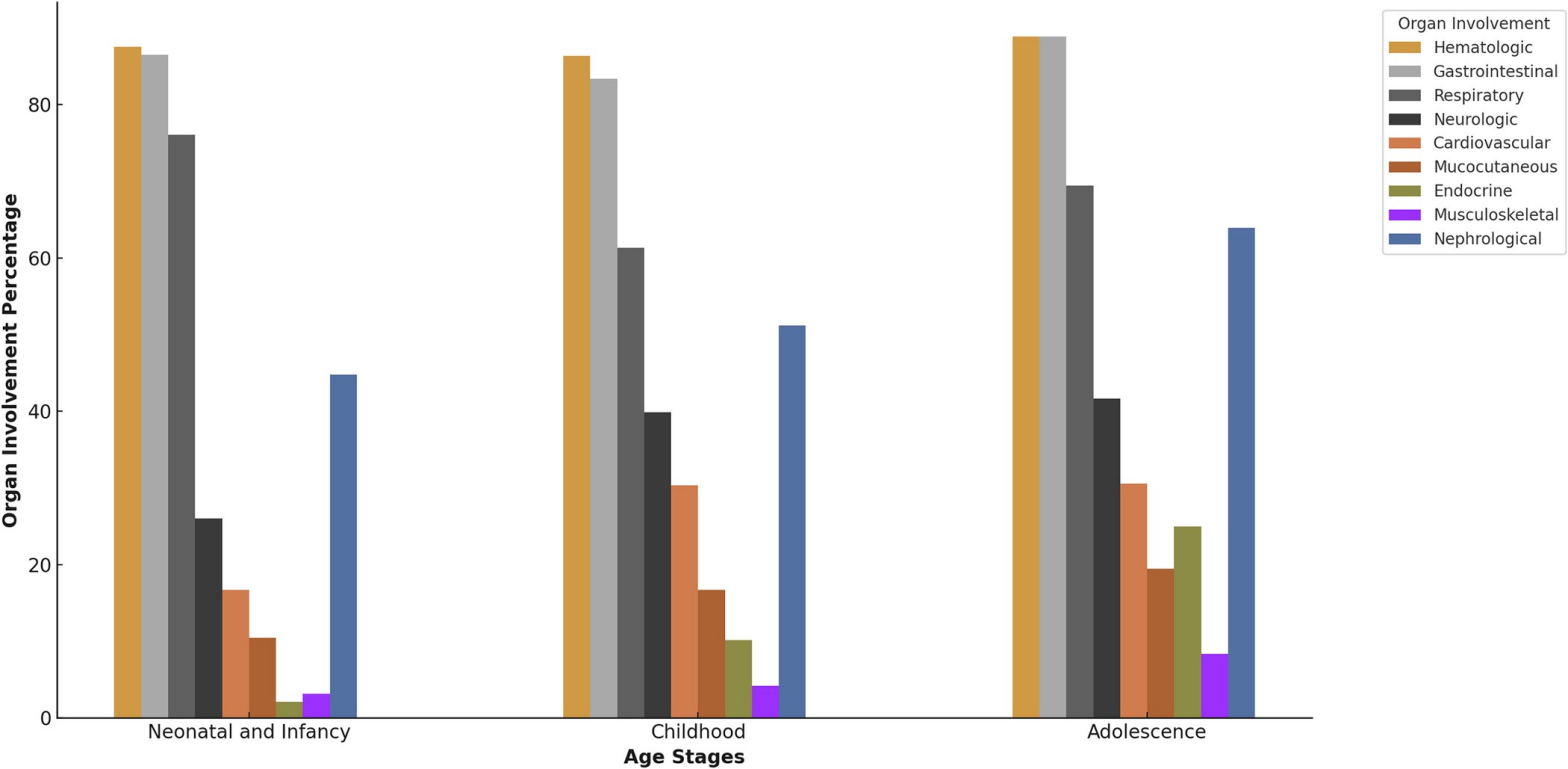
Organ involvement across age stages in MIS-C.

The findings related to organ involvement indicated that the hematological system was the most involved organ system, accounting for 87% of cases. This was followed by gastrointestinal involvement in 85% and respiratory involvement in 67% of patients. The related findings concerning organ involvement are presented in Table 1 (definitions of organ involvement are provided in S1 Table). Also, the percentage of organ involvement across different age groups is shown in Fig 1. Hematologic and gastrointestinal systems show consistently high involvement across all age groups. Except for respiratory involvement, which is higher during the neonatal and infancy stages compared to other age groups, the involvement of other organs increases during childhood and adolescence. A significant increase in endocrine involvement is observed in adolescence. Statistical significance is noted in respiratory, cardiovascular, and endocrine involvement with p-values of 0.047, 0.041, and 0.00026, respectively.

**Table 1:** Involvement of body organs in order of frequency in patients with MIS-C.

Organ System	Frequency	Percentage
Hematologic	261	87%
Gastrointestinal	255	85%
Respiratory	201	67%
Nephrological	152	50.6%
Neurological	107	35.7%
Cardiovascular	78	26%
Mucocutaneous	45	15%
Endocrine	28	9.3%
Musculoskeletal	13	4.3%

The most common gastrointestinal manifestation was nausea and vomiting, followed by diarrhea. The most common respiratory manifestations were cough and shortness of breath. A decrease in blood oxygen saturation was observed in 9 patients (3%).

The most frequent neurological involvement was seizures, seen in 61 patients (20.3%). Symptoms of increased intracranial pressure, such as double vision and cranial nerve VI palsy, were observed in 5 patients (1.7%). One patient (0.3%) also experienced intracranial hemorrhage (ICH) during hospitalization.

A total of 78 patients (26%) had cardiovascular system involvement. The most common cardiac involvement was pericardial effusion, observed in 20 patients (6.7%). There were 16 instances of reduced ejection fraction (EF). Coronary dilation was observed in 4 patients, and microaneurysms in 1 patient. Additionally, 12 patients (4%) developed cardiogenic shock.

Among 28 patients with endocrine system involvement, hyperglycemia was reported in 24 patients (8%), hypoglycemia in 4 patients (1.3%), diabetic ketoacidosis in 7 patients (2.3%), and hypertriglyceridemia in 1 patient (0.3%).

In this study, 45 patients (15%) showed Kawasaki-like features. The most common clinical findings are presented in Table 2 in order of their frequency.

**Table 2:** The most common clinical signs and symptoms in order of frequency in patients with MIS-C.

Symptom	Frequency	Percentage
Fever	300	100%
Nausea and Vomiting	126	42%
Cough	109	36.3%
Diarrhea	83	27%
Seizures	61	20.3%
Shortness of Breath	47	15.6%
Kawasaki-like Features	45	15%
Reduced Appetite	45	15%
Respiratory Distress	37	12.3%
Skin Rash	33	11%
Conjunctivitis	27	9%
Headache	27	9%
Abdominal Pain	26	8.6%

Based on paraclinical findings, 21 patients (7%) had electrolyte disorders, with the most common being hypocalcemia in 8 patients (2.7%).

Abnormal urinalysis was reported for 151 patients (50.3%), among which glucosuria was found in 83 cases (27.7%) and proteinuria in 29 cases (9.7%). Elevated white blood cells were seen in the urine of 39 patients (13%), and red blood cells were found in the urine of 24 patients (8%). Furthermore, 42 patients (14%) had abnormal stool tests, with 27 cases showing white blood cells (9%), 13 cases showing red blood cells (4.3%), and 8 cases showing yeast (2.7%) in the stool.

According to Table 3, elevated D-dimer levels were the most common paraclinical finding, with a frequency of 83.3%. This was followed by increased ferritin in 71.4%, elevated troponin in 66%, elevated ESR in 59.7%, lymphopenia in 51%, and increased CRP in 49.7%.

**Table 3:** The most common laboratory findings in order of frequency in patients with MIS-C.

Finding	Frequency (out of 300)	Percentage
Increased D-dimer	250	83.3%
Increased Ferritin	214	71.3%
Increased Troponin	198	66%
Increased ESR	180	60%
Lymphopenia	153	51%
Increased CRP	150	50%
Decreased Albumin	129	43%
Increased AST	129	43%
Anemia	123	41%
Glucosuria	83	27.7%
Neutrophilia	82	27.3%
Thrombocytosis	62	20.7%
Increased ALT	59	19.7%
Increased LDH	46	15.3%
Leukopenia	46	15.3%
Leukocytosis	39	13%
Sterile Pyuria	39	13%
Thrombocytopenia	36	12%

The outcomes of MIS-C at the end of hospitalization are presented in Table 4. The findings show that 81.7% of patients were hospitalized in isolation throughout the duration, and 18.3% required ICU admission. Overall, 1% of the patients died during hospitalization.

**Table 4:** Outcomes of MIS-C at the end of hospitalization.

Outcome	Frequency	Percentage	Median Duration of Hospitalization (IQR)
Hospitalized in Isolation	245	81.7%	4.41 (2.9–6.5)
ICU Admission	55	18.3%	4.46 (2.6–7.1)
Death	3	1%	-
Recovery	297	99%	-

Based on findings from Table 5, there is a significant association between cardiovascular involvement and all measured variables, including blood platelet levels, ESR, CRP, serum troponin, ferritin, albumin, and D-dimer levels, each showing a P value ≤0.01. Out of 300 patients, 78 patients (26%) had cardiovascular involvement.

**Table 5:** Association of paraclinical findings with cardiovascular involvement due to MIS-C.

Variable		Cardiovascular Involvement		P value
		No Frequency (%)	Yes Frequency (%)	
**Platelet**	Low	20 (9%)	16 (20.5%)	<0.001
	Normal	172 (77.5%)	30 (38.5%)	
	High	30 (13.5%)	32 (41%)	
	Total	222 (100%)	78 (100%)	
**ESR**	Normal	110 (49.5%)	10 (12.8%)	<0.001
	High	112 (50.4%)	68 (87%)	
	Total	222 (100%)	78 (100%)	
**CRP**	Normal	126 (56.7%)	24 (30.7%)	<0.001
	High	96 (43.2%)	54 (69.2%)	
	Total	222 (100%)	78 (100%)	
**Troponin**	Negative	96 (43.2%)	6 (7.7%)	<0.001
	Positive	126 (56.7%)	72 (92.3%)	
	Total	222 (100%)	78 (100%)	
**D-dimer**	Normal	50 (22.5%)	0 (0%)	<0.001
	High	172 (77.5%)	78 (100%)	
	Total	222 (100%)	78 (100%)	
**Ferritin**	Normal	49 (22%)	37 (47.4%)	<0.001
	High	173 (78%)	41 (52.5%)	
	Total	222 (100%)	78 (100%)	
**Albumin**	Low	68 (30.6%)	61 (78.2%)	<0.001
	Normal	154 (69.4%)	17 (21.8%)	
	High	0 (0%)	0 (0%)	
	Total	222 (100%)	78 (100%)	

## Discussion

Herein, we studied the clinical and laboratory characteristics of 300 patients admitted to our hospital with the confirmed diagnosis of MIS-C. Most of the patients in our study belong to the childhood age group, with 168 individuals (56%). The median age of MIS-C patients in the present study was 3 years, which is close to the median ages reported in studies by Buonsenso et al. [18], Abdelaziz et al. [19], Rostami-Maskopaee et al. [20], Karunakar et al. [21], and Kapoor et al. [22], with median ages of 3.93, 4, 4.5, 5, and 5.5 years, respectively. This is while many studies have also reported a median age of 8 years or older [11,23–26]. The difference in the study population’s age range can be one reason for the variation in median age across different studies. Similar to other studies, a higher gender distribution of MIS-C in boys compared to girls was observed in this study as well [11,19–21,23,25,26].

In terms of systemic involvement, hematologic system involvement has been the most prevalent, with a frequency of 87%, making it the most common system affected. Following hematologic involvement, the gastrointestinal, respiratory, nephrologic, neurologic, cardiovascular, mucocutaneous, endocrine, and musculoskeletal systems rank next with frequencies of 85%, 67%, 50.7%, 35.7%, 26%, 15%, 9.3%, and 4.3%, respectively. In a multicenter study by Rostami-Maskopaee et al. in Iran, the highest involvement was in the gastrointestinal and hematologic systems, with frequencies of 88.89% and 84.44%, respectively [20]. Similarly, studies conducted in the United States also showed the highest involvement in the gastrointestinal, cardiovascular, and hematologic systems in that order [11,26]. However, a multicenter study by Mehra et al. in India reported that although the gastrointestinal system had the highest involvement at 96%, hematologic involvement was lower than in our study, at 54% [27].

Overall, the involvement of the gastrointestinal, respiratory, and neurologic systems in this study aligns with that of other studies. However, mucocutaneous and cardiovascular involvement is significantly less than in other studies [11,22,26–28]. Additionally, renal system involvement is higher in this study compared to others [20,24,26,27]. Endocrine manifestations reported in this study include hyperglycemia (8%), hypoglycemia (1.3%), and diabetic ketoacidosis (3.2%), which have not been reported in any epidemiological studies on MIS-C clinical manifestations so far. On the other hand, the study by Saritas Nakip et al. demonstrated that hyperglycemia is prevalent in MIS-C patients [29]. Case reports also mention the occurrence of diabetic ketoacidosis in MIS-C patients [30,31]. The findings of this study regarding the association of MIS-C with the endocrine system can provide valuable insights for physicians in terms of offering necessary therapeutic solutions.

Moreover, the results of our study reveal significant age group-related differences in organ involvement in MIS-C, with consistently high involvement of hematologic and gastrointestinal systems across all age groups. The increased cardiovascular involvement during adolescence compared to the other age groups aligns with the findings of Dufort et al. [32]. Additionally, the marked increase in endocrine involvement during adolescence may be linked to hormonal changes during puberty [33].

Excluding fever, which is a pre-requisite for MIS-C diagnosis, the most prevalent symptoms observed upon presentation include nausea and vomiting (42%), cough (36.3%), diarrhea (27%), seizures (20.3%), shortness of breath (15.6%), decreased appetite (15%), and Kawasaki-like manifestations (15%). In a systematic review by Radia et al., which encompassed 783 MIS-C cases from 35 different studies, vomiting and diarrhea, similar to our research, were among the symptoms with the highest prevalence [34]. However, in contrast to our findings, rashes were the most clinically prevalent symptom with 42% frequency. Additionally, cough was observed in only 4.5% of cases in this study [34]. In the study by Godfred-Cato et al., abdominal pain, vomiting, diarrhea, and rash were the most prevalent clinical symptoms, each with over 50% prevalence. The frequency of cough in this study was 28.6% [26]. While abdominal pain was reported in over 50% of cases in many studies, its prevalence in our study was only 8.6% [24,26,32,35]. In the systematic review by Kaushik et al., Kawasaki-like manifestations were observed in 36% of individuals, more than twice in our study [36].

Echocardiography conducted on 108 individuals (36% of the study population) revealed pericardial effusion, decreased EF, tricuspid valve insufficiency, mitral valve insufficiency, and coronary artery involvement in 18.5%, 14.8%, 14.8%, 13.8%, and 4.6% of cases, respectively. This contrasts with the findings of the study by Rostami-Maskopaee et al., where echocardiographic results in 88.1% of patients showed mitral valve insufficiency, tricuspid valve insufficiency, coronary artery dilatation, pericardial effusion, and decreased EF in 88.7%, 64.1%, 31.3%, 32.3%, and 26.6% of patients, respectively [20].

Among the laboratory findings, the highest abnormal values were related to inflammatory markers. Increases in D-dimer, ferritin, troponin, ESR, CRP, LDH, and a decrease in albumin were observed in 3.83%, 4.71%, 7.66%, 7.59%, 7.49%, 5.15%, and 6.43% of cases, respectively. These results were consistent with previous studies, except for CRP, where previous studies reported a higher percentage increase [20,32]. In this study, blood markers including lymphopenia, anemia, neutrophilia, thrombocytosis, leukopenia, leukocytosis, thrombocytopenia, neutropenia, and lymphocytosis were observed in 51%, 41%, 27.3%, 20.7%, 15.3%, 13%, 12%, 8.7%, and 2% of cases, respectively. These results were in line with the findings of a systematic review, where lymphopenia, neutrophilia, and thrombocytopenia were observed in 58%, 22%, and 22% of individuals, respectively [36]. Among the measured liver enzymes, AST increased in 43%, and ALT increased in 19.7% of cases. The increase in these two enzymes was higher in our study compared to the study by Hajiani Ghotbabadi et al. and was approximately consistent with the study by Kiani et al. [37,38].

Urinalysis of 50.3% of MIS-C patients in this study was abnormal, including glucosuria, sterile pyuria, proteinuria, and RBC in urine, observed in 27.7%, 13%, 9.7%, and 8% of cases, respectively. In most epidemiological studies reporting clinical manifestations of MIS-C, there was no mention of abnormal urinalysis. However, in the study by Ludwikowska et al., leukocyturia and dysuria were observed in 19.4% and 15.6% of cases, respectively [28]. Overall, limited studies have reported glucosuria, sterile pyuria, proteinuria, and RBC in the urine of MIS-C patients [39–41]. The renal involvement and abnormal urinalysis in patients with MIS-C, as also addressed in the study by Meneghel et al., underscores the importance of clinical and laboratory data in the current study [39].

In addition to urinalysis, abnormal stool tests have been recorded in 14% of cases, with the presence of WBC and RBC in the stool being the most common findings at frequencies of 9% and 3.4%, respectively. Apparently, in other epidemiological studies, there have been no reports on the results of stool tests. However, in case report studies, abnormal stool tests have been reported in MIS-C patients [42,43].

Despite the association between inflammatory markers and cardiovascular involvement, their correlation in MIS-C patients with cardiovascular involvement has not been thoroughly investigated [44]. This study found a significant association between cardiovascular involvement and blood platelet levels, ESR, CRP, serum troponin, serum ferritin, D-dimer, and albumin levels. These findings align with previous studies, which have identified cardiovascular disease as a critical biomarker for increased risk and poorer prognosis in COVID-19 patients, highlighting the need to monitor cardiovascular health in infected individuals [5]. Similarly, in the study by Momtazmanesh et al., an increase in the levels of cardiac and inflammatory biomarkers was reported in patients with cardiovascular involvement following COVID-19, which aligns with the current study’s findings [45]. In the study by Whittaker et al., unlike the findings of our research, a significant correlation between inflammatory markers and coronary vessel involvement was not reported [46]. Also, in the study by Zhao et al., there was no significant correlation between cardiac involvement in MIS-C patients and elevated troponin levels [47]. Based on the results of the current study, abnormal levels of blood platelets, ESR, CRP, serum troponin, serum ferritin, D-dimer, and albumin serve as risk factors for cardiovascular involvement in patients diagnosed with MIS-C. Therefore, if these risk factors are observed in MIS-C patients, echocardiography is recommended to assess cardiovascular involvement.

Given the complex inflammatory responses in MIS-C, specific cytokines such as IL-1, IL-6, and TNF-α are critical biomarkers for evaluating disease progression and prognosis [48]. Monitoring increased cytokine levels can facilitate the evaluation of the inflammatory condition, aiding in diagnosis and treatment decisions in MIS-C cases. The association of increased pro-inflammatory cytokines with outcomes in pediatric COVID-19-related syndromes enhances the clinical applicability of cytokine profiling among such patients [48]. Although cytokine levels were not measured in this study, a relationship between the putative role of specific cytokines such as IL-1, IL-6, and TNF-α as biomarkers in estimating the course of the disease and prognosis in MIS-C can be related. Subsequent research may expand upon these findings to evaluate the efficacy of cytokine profiling in forecasting cardiovascular outcomes in MIS-C patients.

The examination of the outcomes of MIS-C in this study reveals that 81.7% of the patients were hospitalized in the isolation ward during the entire hospitalization period, and 18.3% of patients needed hospitalization in the ICU. The duration of hospitalization in isolated wards ranged from 1 to 21 days (with a median of 4.41 days), and in the ICU, it varied from 1 to 17 days (with a median of 4.46). Additionally, 1% of the enrolled patients succumbed during hospitalization. In the study by Choe et al. during the pre-delta variant of the SARS-CoV-2 period, 50% of patients required ICU admission [49]. This percentage dropped to 26.3% and 10.6% during the delta and omicron variant periods, respectively [49]. The median hospitalization days in the study by Godfred-Cato et al. were 6 and 5 for hospital and ICU, respectively [26]. Furthermore, the mortality rate in this study was 1.7%, which is close to our study results [26]. Two systematic reviews with data from 655 and 783 individuals reported mortality rates close to ours [34,36]. The mortality rate reported in Iran, according to the Mamishi et al. study, was 1.6%, which is similar to our findings, whereas Rostami-Maskopaee et al. reported a rate of 4.44% [20,50]. Given the varying disease severity of different SARS-CoV-2 strains, the outcomes of MIS-C may differ across studies due to differences in data collection times. Additionally, the size of the studied population plays a crucial role in accurately determining disease outcomes.

Our study had notable limitations that should be acknowledged. The first limitation is associated with potential inadequacies in medical records. These inadequacies may arise from the challenges inherent in documenting accurate medical histories, particularly in very young patients who may have limited prior medical documentation or are unable to communicate their symptoms effectively. Moreover, the socioeconomic background of patients and potential biases from diagnosing clinicians could also contribute to incomplete or biased data. The second limitation is the lack of additional investigations, including echocardiography, in a subset of patients. The reasons for this include the absence of confirmation of the final diagnosis or patient discharge with personal consent before the scheduled time. Furthermore, the inherent limitation in studying pediatric populations is the difficulty in ensuring that the children’s voices are heard and considered in both medical and research contexts. Often, children are represented by others who speak on their behalf, which can lead to a lack of firsthand input from the patients themselves regarding their experiences and symptoms.

## Conclusion

We have presented an experience with distinct clinical and laboratory manifestations in MIS-C. The median age of MIS-C patients in this study was 3 years, and the disease was more predominant in boys. Given the lower median age in this study compared to previous studies, reporting clinical and laboratory manifestations of MIS-C in pediatrics with a younger age is valuable for the diagnosis and treatment course. The hematologic, gastrointestinal, and respiratory systems are the most commonly involved systems in this disease. Endocrine manifestations, including hyperglycemia and diabetic ketoacidosis, were also observed in MIS-C patients. Renal involvement and abnormal urinalysis were significantly prevalent in MIS-C patients and should receive more attention. Nausea and vomiting, cough, diarrhea, seizures, shortness of breath, decreased appetite, and Kawasaki-like symptoms were the most common clinical symptoms in patients. Elevated D-dimer, ferritin, troponin, ESR, and lymphopenia were the most common laboratory findings in MIS-C. Disturbances in liver enzymes, especially AST, were also evident. Platelet disorders and abnormal levels of ESR, CRP, serum troponin, serum ferritin, D-dimer, and albumin were risk factors for cardiovascular involvement, and consequently, echocardiography is recommended in MIS-C patients with these laboratory indications. Out of 300 hospitalized patients in this study, 18.3% required admission to the ICU, and the mortality rate was 1%. Since the emergence of MIS-C, physicians have faced challenges in various manifestations of MIS-C that can lead to misdiagnosis. Due to the unique clinical manifestations of MIS-C involving multiple systems and the difficulty in obtaining an accurate epidemiological history, the diagnosis of MIS-C requires special attention. Given the lack of a specific diagnostic test for this emerging disease, studies focusing on the investigation of clinical symptoms and findings are valuable.

## Supporting information

S1 Table Definitions and observed findings of organ involvement in MIS-C patients. (DOCX)

S1 File Raw data of the study. (XLSX)
