# Supplementary material for: Evaluation of clinical and laboratory findings in MIS-C patients associated with COVID-19: An experience from the Northwest of Iran
Source: PLoS One. 2024 Nov 21;19(11):e0313843. doi: 10.1371/journal.pone.0313843 (PMC11581280; doi:10.1371/journal.pone.0313843)
Supplement: S1 Table — (DOCX) [file pone.0313843.s001.docx]

**S1 Table. Definitions and Observed Findings of Organ Involvement in MIS-C Patients.**

| **System Organ Involvement** | **Definition of Involvement** | **Observed Findings** |
| --- | --- | --- |
| Hematologic | Any signs of involvement of blood cell lines observed in laboratory tests | Abnormal values of white blood cells (WBC), neutrophils (ANC), lymphocytes (ALC), platelets (PLT), and hemoglobin (Hb) |
| Gastrointestinal | Any signs of gastrointestinal involvement, including diarrhea, vomiting, and abdominal pain | Diarrhea, Nausea/Vomiting, Anorexia, Abdominal pain, Hematemesis, Dysentery, Icterus, Constipation |
| Respiratory | Any signs of respiratory system involvement, including cough, shortness of breath, or abnormal findings in chest X-ray | Cough, Productive cough, Dyspnea, Respiratory distress, Cyanosis, Chest pain, Decreased oxygen saturation, Crackles, Wheezing, Abnormal chest X-ray findings, Abnormal CT findings |
| Nephrological | Abnormalities in urinalysis or elevated creatinine levels | Glucosuria, Proteinuria, Elevated creatinine, Abnormal urinalysis with negative urine culture (WBC, RBC, WBC clumps, Positive nitrite) |
| Neurological | Any signs of nervous system involvement, including headache and seizures | Seizure, Headache, Vertigo, Loss of consciousness, Increased intracranial pressure, Paresthesia, Intracerebral hemorrhage |
| Cardiovascular | Any signs of cardiovascular system involvement, including palpitations, chest pain, heart murmur, or abnormal findings in ECG or echocardiography | ECG changes (Bradycardia, Tachycardia, Block, PSVT, Long QT, ST-T changes), Decreased ejection fraction, Right ventricular dysfunction, Right ventricular hypertrophy, Left ventricular hypertrophy, Dilated cardiomyopathy, Mitral regurgitation, Tricuspid regurgitation, Aortic insufficiency, Pulmonary hypertension, Mild pericardial effusion, Coronary artery abnormalities (microaneurysm, coronary shining, RCA ectasia, LMC ectasia) |
| Mucocutaneous | Any signs of skin or mucous membrane involvement, including rash, desquamation, and oral ulcers | Rash, Edema, Bilateral conjunctivitis, Strawberry tongue, Lips and oral cavity lesions, Desquamation, Itching, Cervical lymphadenopathy |
| Endocrine | Any signs of endocrine system involvement, including hypo- or hyperglycemia | High blood sugar, Diabetic ketoacidosis (DKA), Hypoglycemia, Hypertriglyceridemia, Electrolyte disorders (Hypernatremia, Hyponatremia, Hyperkalemia, Hypokalemia, Hypercalcemia, Hypocalcemia, Hypermagnesemia, Hypomagnesemia, Hypophosphatemia) |
| Musculoskeletal | Any signs of musculoskeletal system involvement, including myalgia and arthralgia. | Myalgia, Arthralgia, Hypotonia (in infants) |
